# Edible Insects for Future Protein and Product Development: Mapping Global Research Trends, Innovation, and Development Through Bibliometric Analysis

**DOI:** 10.3390/insects17101005

**Published:** 2026-09-30

**Authors:** Dewi Anggraini, Eny Palupi, Irma Isnafia Arief, Uswatun Hasanah, Anuraga Jayanegara, Muhammad Rizal Martua Damanik, Rimbawan Rimbawan, Ade Chandra Iwansyah, Rizal Bahara

**Affiliations:** 1 Magister Study Program in Nutrition Science, Faculty of Medicine and Nutrition, IPB University, Bogor 16680, Indonesia; 2 Division of Food Management and Environmental Health, Faculty of Medicine and Nutrition, IPB University, Bogor 16680, Indonesia; 3 Department of Animal Production and Technology, Faculty of Animal Science, IPB University, Bogor 16680, Indonesia; 4 Division of Food Science and Technology, Faculty of Engineering and Technology, IPB University, Bogor 16680, Indonesia; 5 Department of Nutrition and Feed Technology, Faculty of Animal Science, IPB University, Bogor 16680, Indonesia; 6 Division of Clinical Nutrition, Faculty of Medicine and Nutrition, IPB University, Bogor 16680, Indonesia; 7 Research Center for Food Technology and Processing, National Research and Innovation Agency (BRIN), Jl. Jogja–Wonosari Km. 31.5, Gunungkidul, Yogyakarta 55861, Indonesia; 8 Natural Resources and Environmental Management Science Study Program, Graduate School, IPB University, Bogor 16680, Indonesia

**Keywords:** entomophagy, insect protein functionality, consumer acceptance, food safety, bibliometric mapping

## Abstract

The increasing demand for sustainable protein sources has stimulated growing scientific interest in edible insects as a promising component of future food systems. This study aimed to map the global research landscape and thematic development of edible insect-based product research. A bibliometric analysis was conducted using a Scopus-indexed dataset of 542 records (536 records classified by Scopus as Articles and 6 as Conference Papers) published from 2016 to 2025. Keyword co-occurrence analysis (Author + Index Keywords, minimum occurrence threshold = 5, *n* = 317 keywords and 14,221 co-occurrence links, with an explicit thesaurus applied to merge terminology variants) identified seven thematic clusters: (1) entomophagy, novel food, and consumer safety; (2) nutritional and compositional validation; (3) protein structural and functional properties; (4) taxon-specific protein sources; (5) bioactive compounds and enzymatic processing; (6) allergenicity and immunological safety; and (7) taxonomic and chemical characterization. Publications increased 16.6-fold between 2016 and 2025 (CAGR ≈ 36.6% year^−1^), led by South Korea (66 documents), Italy (53), China (48), the United States (41), and Poland (33). Mean citations per document varied across publication cohorts but generally declined toward the most recent years because of unequal citation windows, from 115.1 (2016) to 1.7 (2025), with a higher value in 2017 (195.9). Country-level network analysis shows the United States, Germany, and Italy occupy the strongest structural bridging positions, while South Korea, despite leading in publication volume, shows comparatively limited international-collaboration integration. Key gaps include the underrepresentation of Africa and Southeast Asia in the Scopus-indexed international record, limited independent allergenicity and immunological-safety vocabulary, and scarce techno-economic and large-scale processing evidence. These findings identify priorities for strengthening the safety, technological development, consumer relevance, and international collaboration of insect-based food research.

## Simple Summary

Edible insects are increasingly studied as a sustainable protein source and food ingredient for the future. This study examined 542 Scopus-indexed records published between 2016 and 2025 to understand how research on edible insects for food products has grown and changed over time. We found that publication output increased more than sixteen-fold over the decade, with South Korea, Italy, China, the United States, and Poland among the leading contributors. Seven main research areas were identified, covering nutritional and compositional validation, protein structure and functionality, entomophagy and consumer-related research, taxon-specific protein sources, bioactive compounds and enzymatic processing, allergenicity and immunological safety, and taxonomic and chemical characterization. Research has diversified from foundational nutritional and compositional studies toward more applied topics involving food processing, consumer acceptance, safety, and product innovation. Although South Korea had the highest publication volume, its international collaboration network was comparatively limited, whereas the United States, Germany, and Italy occupied stronger bridging positions. Africa and Southeast Asia were less visible in the Scopus-indexed international record. These findings help identify research and collaboration priorities for the safe and sustainable development of insect-based foods.

## 1. Introduction

The global food system is experiencing increasing pressure due to population growth, environmental degradation, and the urgent need to transition toward more sustainable protein sources [1,2]. Conventional livestock production remains a major contributor to greenhouse gas emissions, land conversion, and freshwater use, intensifying the search for alternative protein systems with lower environmental burdens [3,4]. Within the broader protein transition discourse, edible insects have emerged as a promising component of sustainable food systems due to their high feed conversion efficiency, reduced ecological footprint, and potential integration into circular bioeconomy models [5,6,7,8,9,10,11].

Edible insects provide high-quality protein, essential amino acids, lipids, micronutrients, and bioactive compounds, supporting their application in both food and feed sectors [12,13]. As a result, research in this field has expanded rapidly across disciplines, including nutrition science, food and feed processing technology, allergenicity assessment, sustainability evaluation, consumer behavior, and regulatory science.

However, the diversification of research domains has generated a complex and increasingly fragmented scientific landscape, and this fragmentation is not merely descriptive: it has direct commercial consequences. Persistent challenges related to allergenic potential, safety standards, regulatory harmonization, and consumer acceptance continue to influence commercialization trajectories [14,15,16], meaning that scientific effort which is unevenly distributed across these bottleneck areas can itself become a barrier to bringing insect-based products to market. This raises a specific empirical question that this bibliometric analysis is designed to answer: is the volume and thematic distribution of published research proportionate to the known commercialization bottlenecks (safety/allergenicity, regulation, and consumer acceptance), or are research resources concentrated disproportionately on compositional and nutritional validation relative to these applied constraints? Simultaneously, technological innovations such as protein extraction, enzymatic hydrolysis, and fermentation are reshaping research directions toward functional and value-added food development [17,18].

In rapidly evolving multidisciplinary fields, bibliometric analysis offers a structured and quantitative approach to map scientific output, thematic evolution, and collaboration networks [19,20]. Through science mapping techniques, bibliometric methods enable the identification of dominant themes, emerging clusters, and structural relationships among authors, institutions, and countries [21]. Although previous bibliometric studies have examined edible insect research, most have primarily focused on publication trends and citation performance, with limited exploration of thematic clustering, collaboration intensity, and knowledge connectivity within the broader sustainable protein transition context [22,23]. Consequently, a more comprehensive and structurally analytical mapping of the field remains warranted.

Accordingly, this study examined the evolution of global scientific output on edible insect-based products over the last 10 years (2016–2025), identified leading contributors (countries, institutions, authors, and journals), mapped major thematic clusters and collaboration networks, and characterized temporal changes in research topics. By integrating publication-performance indicators with science-mapping techniques, this bibliometric assessment clarifies the structural development of the field, identifies knowledge gaps, and informs future research directions within the sustainable protein transition framework.

Relative to prior bibliometric work on edible insects, the present study’s specific contribution is threefold. Recent bibliometric studies have examined the edible insect literature more broadly, providing valuable evidence of the field’s overall growth and citation performance [22,23]. Building on this body of work, the present study complements these efforts by focusing specifically on edible insect-based food and product development within a defined 2016–2025 corpus, allowing closer examination of the thematic and applied dimensions relevant to product innovation. First, it captures the post-2019 acceleration phase, a period not yet fully reflected in earlier bibliometric syntheses. Second, it applies an explicit terminology-normalization procedure prior to constructing a seven-cluster keyword co-occurrence structure. Third, it complements network visualization with quantitative country-level collaboration-network centrality metrics. Together, these analyses provide a combined view of thematic development and international knowledge connectivity within this specific research domain. These contributions are analytical extensions within the fixed 542-record corpus and are not intended to represent comprehensive coverage of the broader edible insect literature.

## 2. Materials and Methods

**Figure 1:**
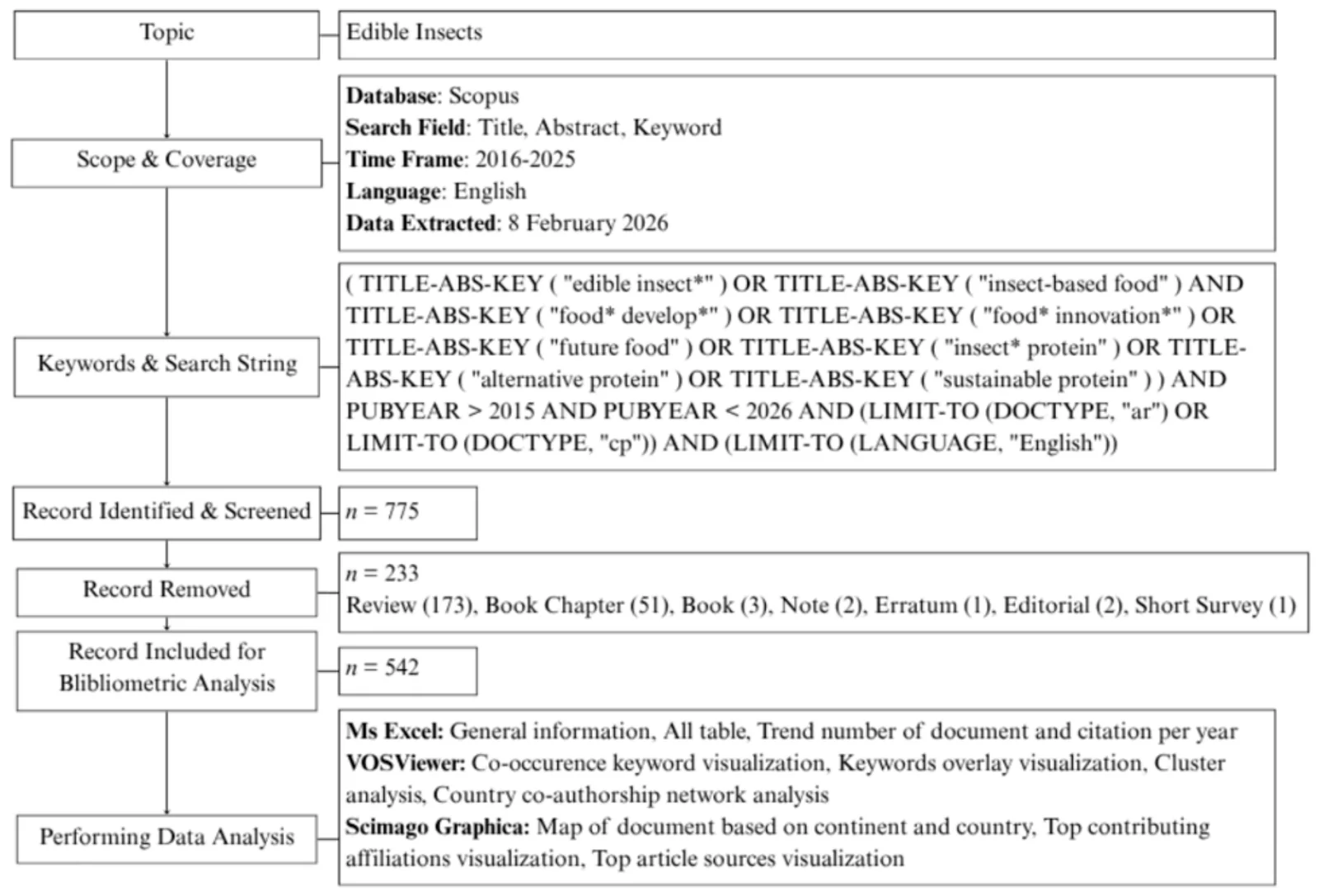
PRISMA-informed reporting workflow for database retrieval and data checking. The 542 records were retained as the bibliometric corpus; post hoc record-level topical verification assessed retrieval risk and did not remove records from the corpus.

This study applied a quantitative bibliometric approach to examine the evolution, intellectual structure, and collaboration patterns of global research on edible insect-based food and product development. Bibliometric analysis offers a systematic framework for assessing scientific performance, thematic development, and knowledge networks in multidisciplinary domains [19,20]. The study used a five-stage workflow for transparent reporting: formulation of search terms, database retrieval, data checking, data processing, and bibliometric/network analysis. Figure 1 shows the PRISMA-informed reporting workflow used to document these stages. The workflow is presented in a PRISMA-informed sequence for transparency, but it should not be interpreted as a systematic-review screening process. The 542 retrieved records were retained as the bibliometric corpus throughout the analysis rather than being reduced through a conventional eligibility filter.

Scopus was selected as the data source because of its broad multidisciplinary coverage of agri-food, nutrition, and food-technology journals relevant to edible insect research, together with structured citation indexing suited to bibliometric extraction. Only English-language records were retained. The Scopus Document Type field classified the corpus as 536 Articles and 6 Conference Papers. No independent dual-author manual title/abstract screening was conducted, and no separate manual eligibility filter was applied after retrieval. Data checking instead focused on the integrity of the exported records and terminology relevant to the bibliometric analyses. Scopus-only and English-only coverage may also underrepresent regional and non-English research, particularly in Africa, Southeast Asia, and other insect-consuming regions.

The search was conducted in the Title, Abstract, and Keywords fields on 8 February 2026 using the Boolean query reproduced below. The parenthesized form shown here was the syntax used for the original retrieval that generated the submitted 542-record corpus. Because the expression contains mixed OR and AND operators within the topical block, the explicit parentheses are important for applying the publication-year, document-type, and language filters to the full topical expression.

(TITLE-ABS-KEY (“edible insect*”) OR TITLE-ABS-KEY (“insect-based food”) AND TITLE-ABS-KEY (“food* develop*”) OR TITLE-ABS-KEY (“food* innovation*”) OR TITLE-ABS-KEY (“future food”) OR TITLE-ABS-KEY (“insect* protein”) OR TITLE-ABS-KEY (“alternative protein”) OR TITLE-ABS-KEY (“sustainable protein”)) AND PUBYEAR > 2015 AND PUBYEAR < 2026 AND (LIMIT-TO (DOCTYPE, “ar”) OR LIMIT-TO (DOCTYPE, “cp”)) AND (LIMIT-TO (LANGUAGE, “English”))

A post hoc record-level topical verification was subsequently conducted in Microsoft Excel to assess topical relevance and potential retrieval risk. The verification used a controlled vocabulary applied to titles, abstracts, author keywords, and index keywords. All 542 records contained at least one insect-related term. A more conservative acceptance rule requiring at least one insect-food term and at least one food/product term was then applied. Under this criterion, 518 of the 542 records (95.6%) met the criterion, whereas 24 records (4.4%) did not. The verification was used to assess retrieval risk rather than as an eligibility filter; therefore, the 24 records were retained in the bibliometric corpus, as failure to match the controlled vocabulary did not by itself establish topical irrelevance. The controlled vocabulary, acceptance rule, and record-level audit are documented in Supplementary File. The original 542-record corpus was therefore retained unchanged for comparability with the submitted analysis.

The time frame (2016–2025) was chosen to capture recent developments over the past decade. Data extraction was performed on 8 February 2026, yielding *n* = 542 records used for all analyses reported below.

### Bibliometric Network Analysis Parameters

Keyword co-occurrence, keyword-overlay, and country co-authorship networks were constructed from the combined Author Keywords and Index Keywords fields of the Scopus export (525 and 263 of 542 records, respectively, contained at least one non-missing keyword field). Prior to network construction, an explicit thesaurus was applied to merge terminology variants, including singular/plural pairs (e.g., “fatty acid”/“fatty acids,” “amino acid”/“amino acids,” “protein”/“proteins,” “peptide”/“peptides”), spelling and hyphenation variants, and targeted synonym pairs (e.g., “consumer behavior”/“consumer behaviour,” “nutritive value”/“nutritional value,” “house cricket”/“Acheta domesticus,” “black soldier fly”/“Hermetia illucens”). Clinical/procedural index terms with no thematic discriminating value (e.g., “controlled study,” “nonhuman,” “animal experiment” as a bare methodological tag, “priority journal”) were removed as stop-terms [24].

Full counting was used, meaning that each keyword pair contributed once per document in which the pair co-occurred. A minimum occurrence threshold of 5 documents was applied, yielding 317 keywords eligible for network construction out of 3703 distinct normalized terms, connected by 14,221 co-occurrence links. Raw co-occurrence counts were used as edge weights; association-strength normalization was not applied. VOSviewer clustering was used with a resolution parameter of 1.15. No additional minimum cluster-size criterion was imposed after clustering; all clusters generated by the software were retained. No additional removal of disconnected or weakly connected keywords was performed beyond the stated occurrence threshold and stop-term procedure.

Microsoft Excel for Microsoft 365 (Microsoft Corporation, Redmond, WA, USA) was used to organize the Scopus export and calculate annual document and citation trends and to conduct the record-level topical verification. VOSviewer version 1.6.20 (Centre for Science and Technology Studies, Leiden University, Leiden, The Netherlands) was used directly for keyword co-occurrence, keyword-overlay, and country co-authorship network analyses and for the native clustering procedure; SCImago Graphica version 1.0.53 (SCImago Lab, Granada, Spain) was used for geographic and affiliation visualizations. No programming-language-based reconstruction of the VOSviewer networks was used.

## 3. Results

### 3.1. Growth and Impact of Edible Insect-Based Product Research (2016–2025)

**Figure 2:**
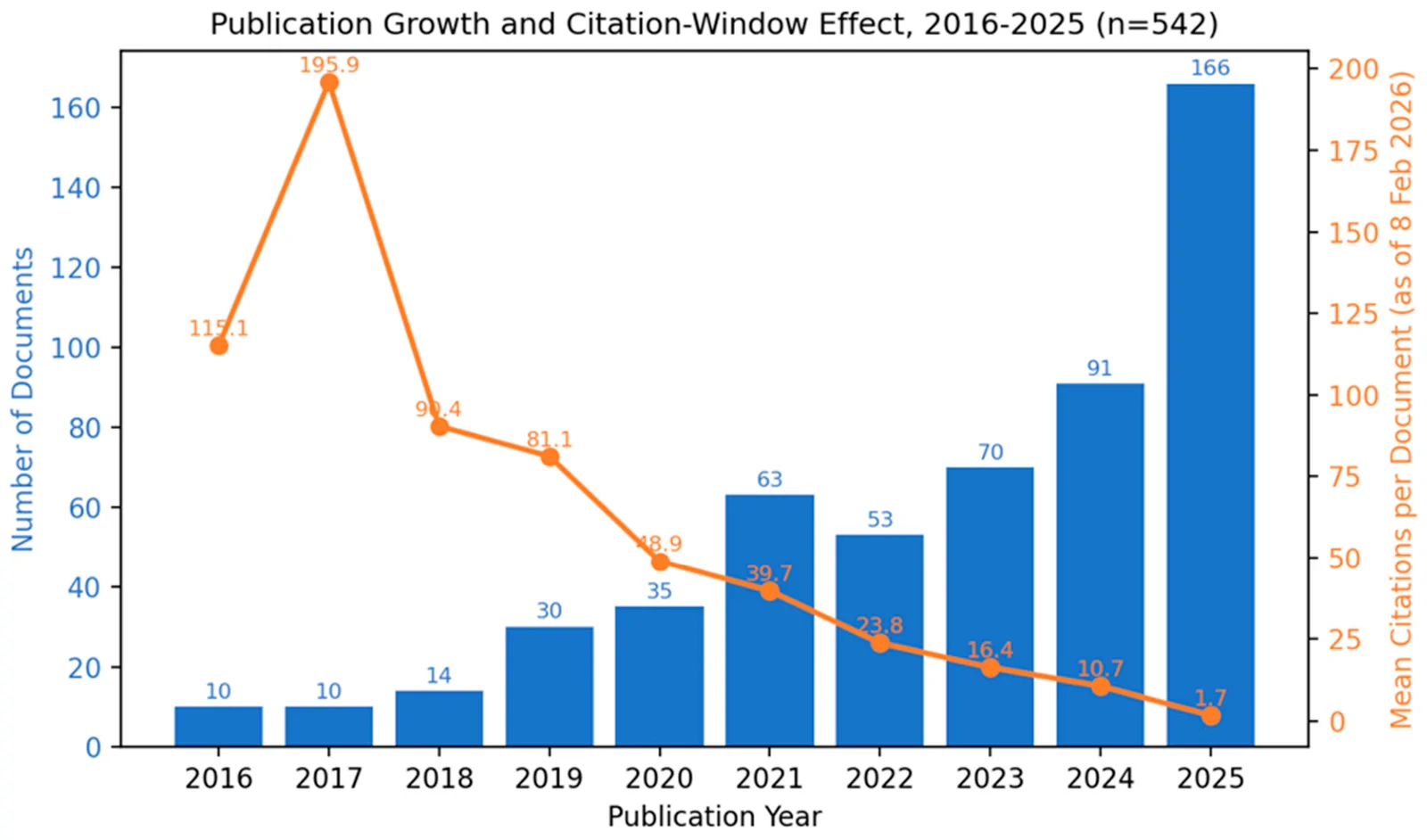
Annual publication output and mean citations per document by publication-year cohort, 2016–2025 (*n* = 542). Bars show documents published per year. The line shows total citations accrued by papers in each publication-year cohort divided by the number of papers in that cohort, calculated from Scopus “Cited by” counts as of 8 February 2026; values therefore reflect unequal citation windows and are not citations received during the corresponding calendar year.

Figure 2 illustrates the temporal evolution of scientific output on edible insect-based product development over the past decade. Publications increased from 10 documents in 2016 to 166 documents in 2025, a 16.6-fold increase, corresponding to a compound annual growth rate (CAGR) of approximately 36.6% per year (10, 10, 14, 30, 35, 63, 53, 70, 91, and 166 documents for 2016–2025, respectively, summing to the full dataset of *n* = 542, verified directly against the underlying export). A particularly sharp increase is observed after 2019, suggesting an acceleration phase likely associated with the growing global emphasis on sustainable food systems and alternative protein sources [1,2].

From a bibliometric perspective, this pattern reflects a transition from an initial emergence phase toward a stage of rapid expansion, characterized by increasing research participation across countries, broader thematic diversification, and stronger integration into global scientific discourse [23,25,26].

In contrast to publication growth, mean citations per document vary substantially across publication-year cohorts and show a general decline toward the most recent years: 115.1 citations per document for the 2016 cohort, 195.9 (2017), 90.4 (2018), 81.1 (2019), 48.9 (2020), 39.7 (2021), 23.8 (2022), 16.4 (2023), 10.7 (2024), and 1.7 for the 2025 cohort, as of the 8 February 2026 extraction date. The higher value for 2017 and the subsequent decline illustrate that citation accumulation is strongly affected by publication age. Earlier cohorts have had substantially more time to receive citations, so raw or mean citation totals by publication year are not directly comparable as measures of relative scientific impact. Accordingly, the observed pattern should not be interpreted as evidence that field influence has declined. Publication volume is the more appropriate indicator of expansion within this observation window, whereas time-normalized or field-normalized citation metrics would be required for cross-cohort impact comparisons [27,28].

### 3.2. Leading Countries, Institutions, Authors, and Journals in Edible Insect Research

#### 3.2.1. Country-Level Contributions

**Figure 3:**
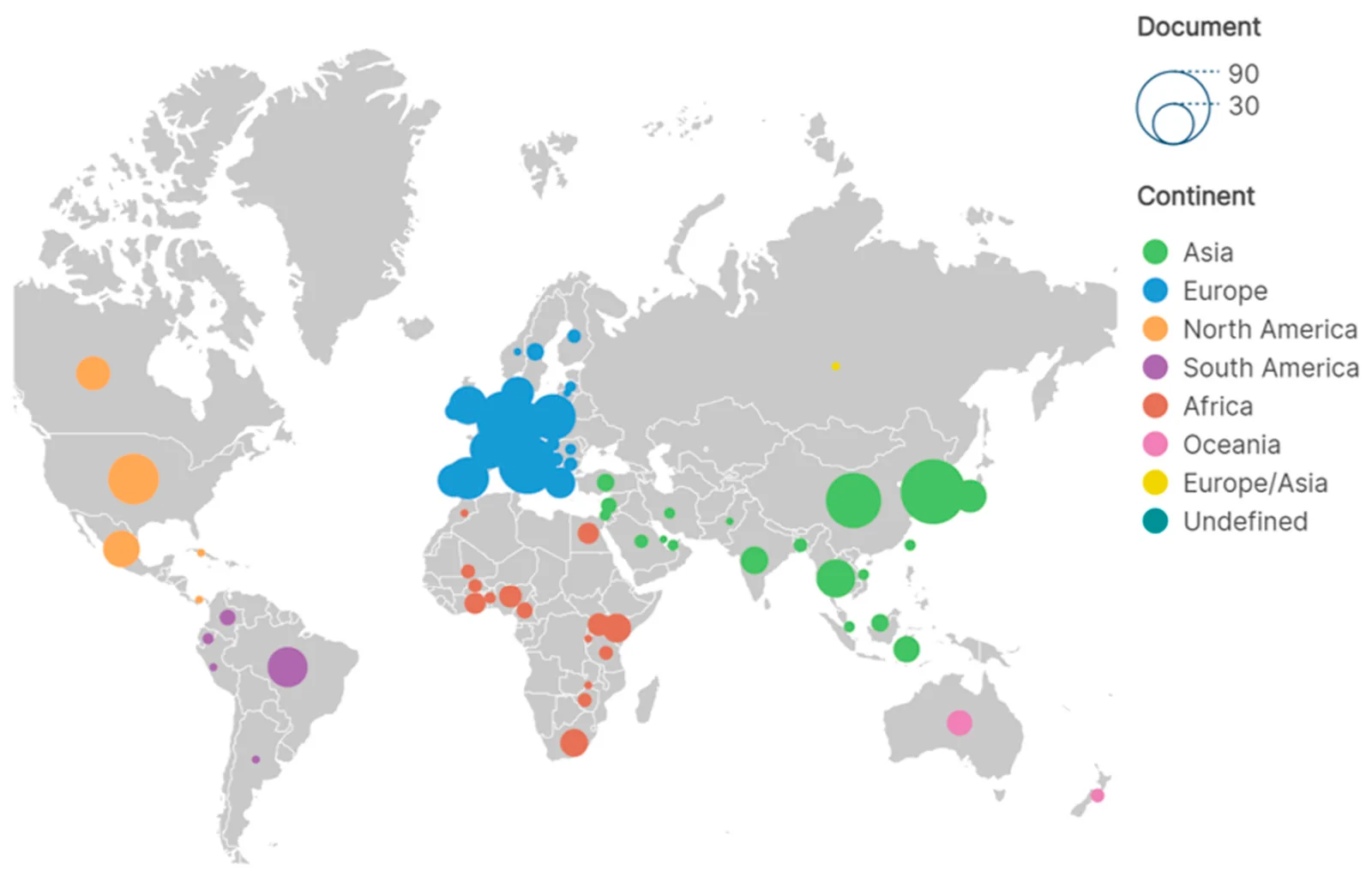
Number of documents about edible insects for product development, by continent. Colors indicate the continent categories shown in the map legend.

Figure 3 shows the geographical distribution of research output by continent. The results reveal a highly uneven yet structurally patterned global landscape. Europe emerges as a dominant research hub, characterized by dense publication activity and extensive inter-country collaboration. This pattern is consistent with the region’s established regulatory and research infrastructure, including the European Union Novel Food framework [15,29], but the bibliometric analysis does not test regulatory policy as a causal determinant of publication output.

Asia represents another major center of research activity, with substantial contributions from South Korea, China, and Thailand. Traditional entomophagy, established research institutions, and national investment in food and biotechnology may all contribute to this visibility [6,30,31]. North American output, particularly from the United States, includes research on product innovation, sustainability, and consumer acceptance [32,33]. These contextual explanations are interpreted cautiously because the present analysis measures indexed publication activity rather than funding levels, local production capacity, or societal relevance [34,35,36].

**Figure 4:**
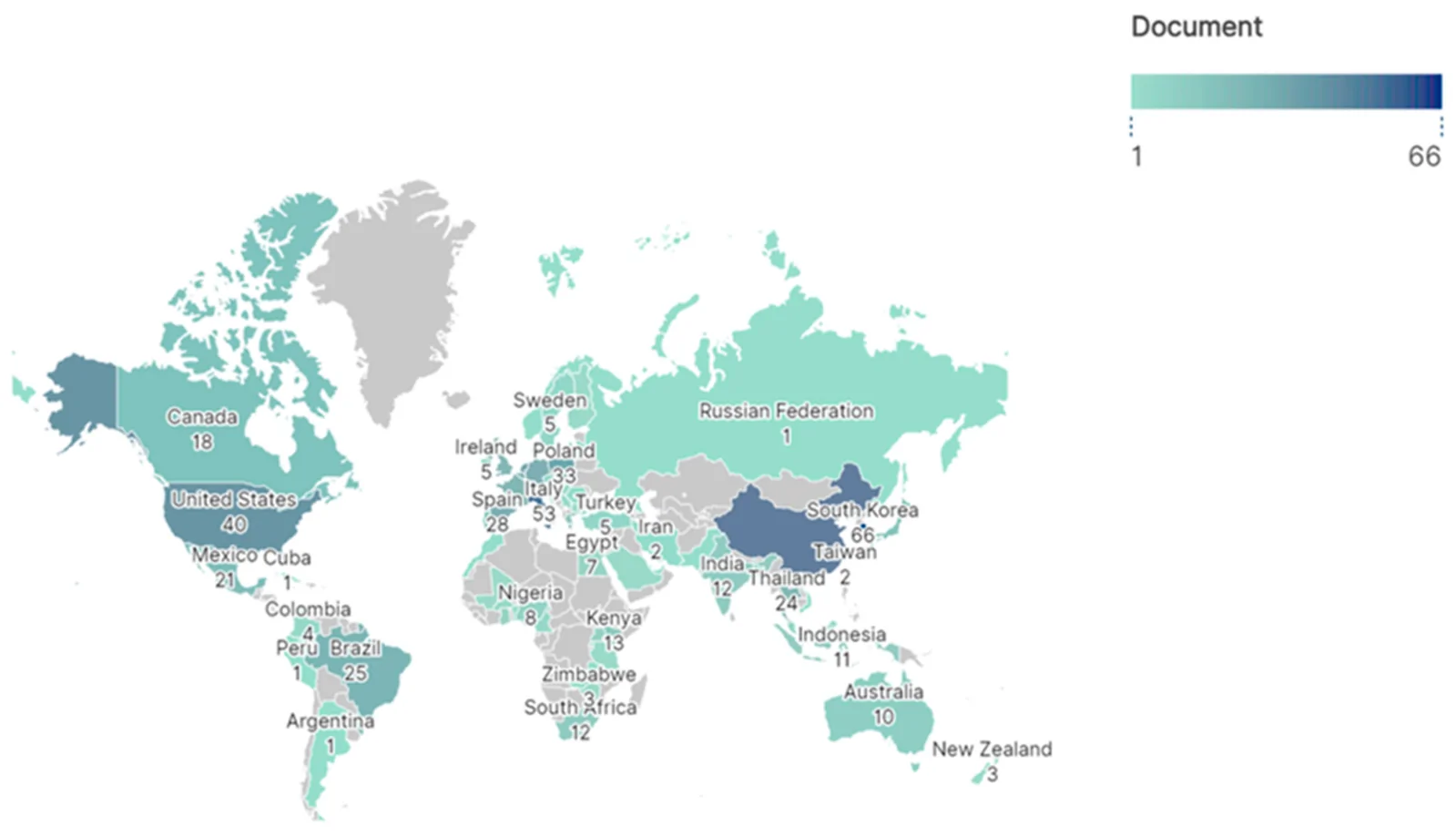
Number of documents on edible insects for product development, by country.

Figure 4 shows the country-level distribution of publication output. South Korea emerges as the leading contributor (66 documents), followed by Italy (53), China (48), United States (41), Poland (33), Netherlands (32), Germany (28), Spain (27), and a tie between Brazil and Belgium (25 each). South Korea’s high output is consistent with documented national support for insect-related food research and farming [37,38], but its relatively low international-network integration is demonstrated more directly by the collaboration metrics in Section 3.4. European countries such as Italy and Poland show substantial activity across processing, food safety, and consumer-oriented studies [4,39]. These observations describe publication patterns and should not be equated with overall national research capacity or commercial activity. Although Africa and South America contribute fewer Scopus-indexed publications, the literature includes research addressing biodiversity utilization, traditional knowledge, food security, and local insect-food systems [40,41,42,43]. Lower indexed output should therefore not be interpreted as evidence of lower local relevance or research activity.

#### 3.2.2. Key Institutions and Authors

**Figure 5:**
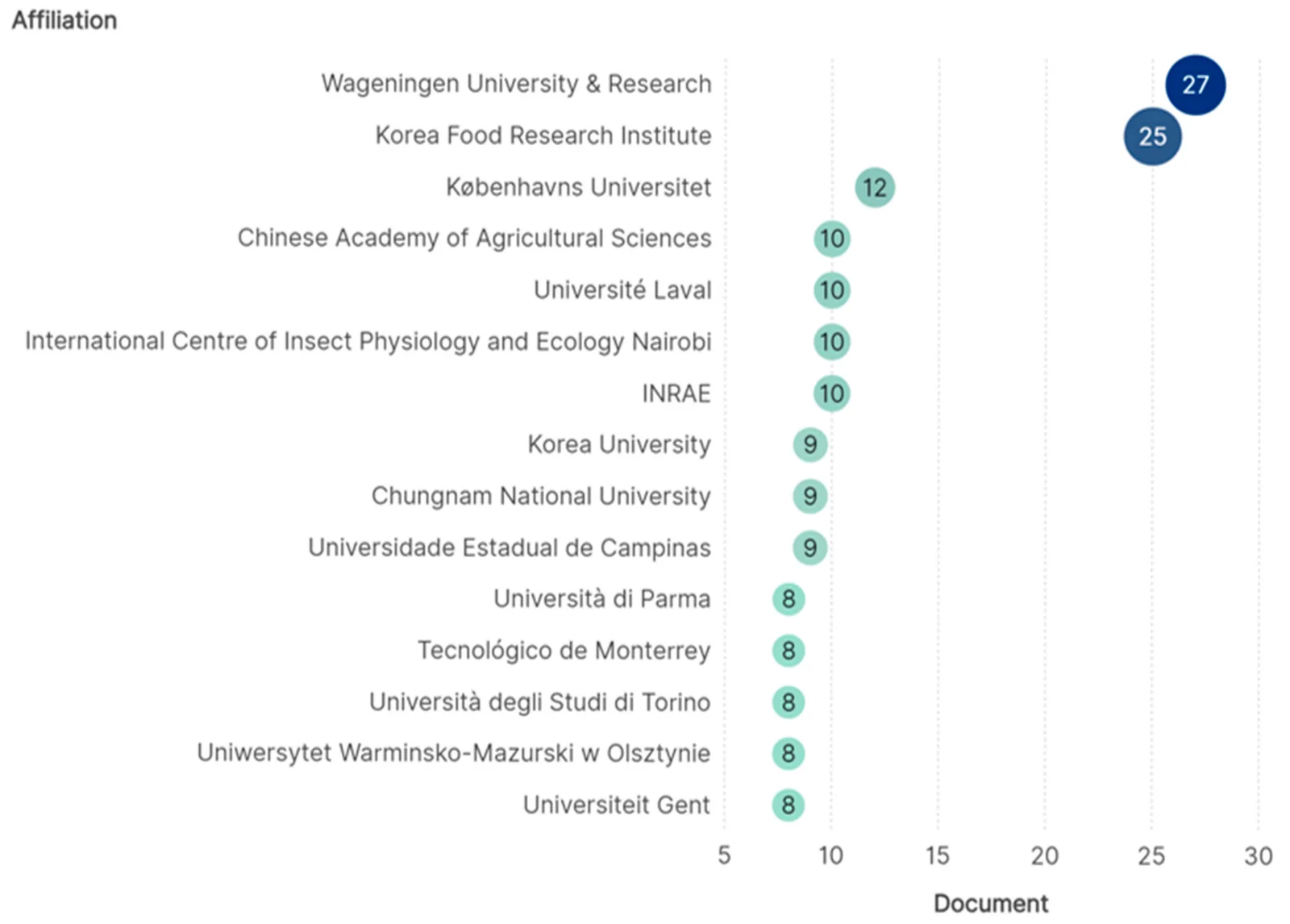
Top contributing affiliations in edible insect-based product development research, ranked by document count. Colors are used for visual distinction only and do not represent an additional variable.

Figure 5 shows the leading contributing affiliations by document count. Institutional output is led by Wageningen University & Research (27 documents) and Korea Food Research Institute (25 documents), followed by a cluster of European, Asian, and Latin American institutions each contributing 7–12 documents.

Table 1 summarizes the ten most productive authors and their citation performance. The leading authors are concentrated within two research groups: Choi, Yun-sang and colleagues at the Korea Food Research Institute, and Fogliano, Vincenzo and Lakemond, Catriona M.M. at Wageningen University & Research. Notably, Fogliano and Lakemond record the highest total citation counts among the top-10 authors (1033 and 1007, respectively) despite lower document counts than the most prolific individual author, indicating disproportionately high per-paper impact from this research group.

#### 3.2.3. Core Journals in Edible Insect Research

**Figure 6:**
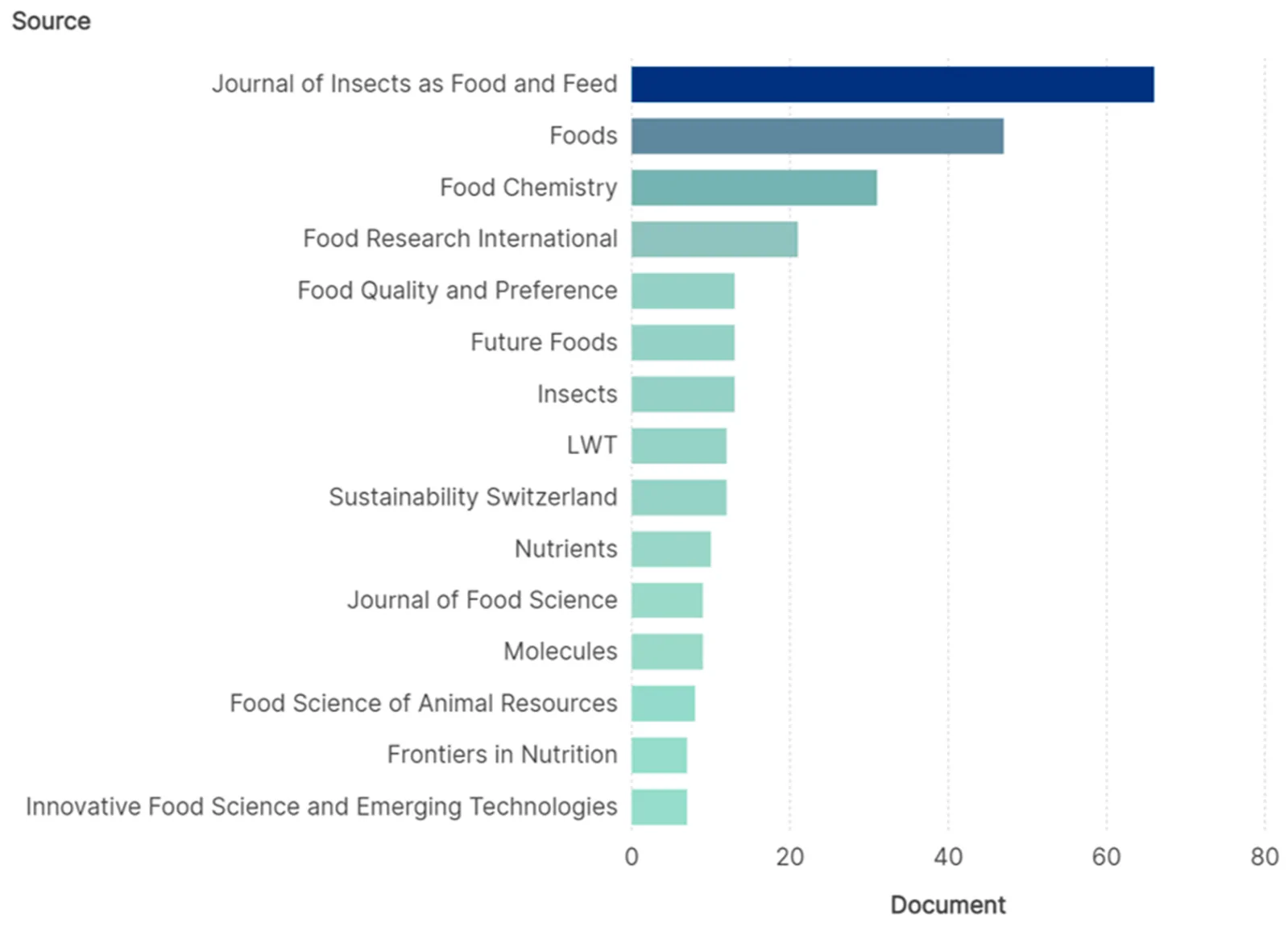
Top publication sources (journals) in edible insect-based product development research, ranked by document count. Colors are used for visual distinction only and do not represent an additional variable.

Figure 6 shows the distribution of publication sources by journal. The Journal of Insects as Food and Feed is the most dominant publication outlet in edible insect-based food research, followed by Foods, Food Chemistry, and Food Research International, reflecting the consolidation of a specialized scholarly platform dedicated to entomophagy, insect production systems, and protein valorization alongside a broader normalization of insect-derived ingredients within high-impact, general food-science journals.

### 3.3. Research Themes and Knowledge Clusters

**Figure 7:**
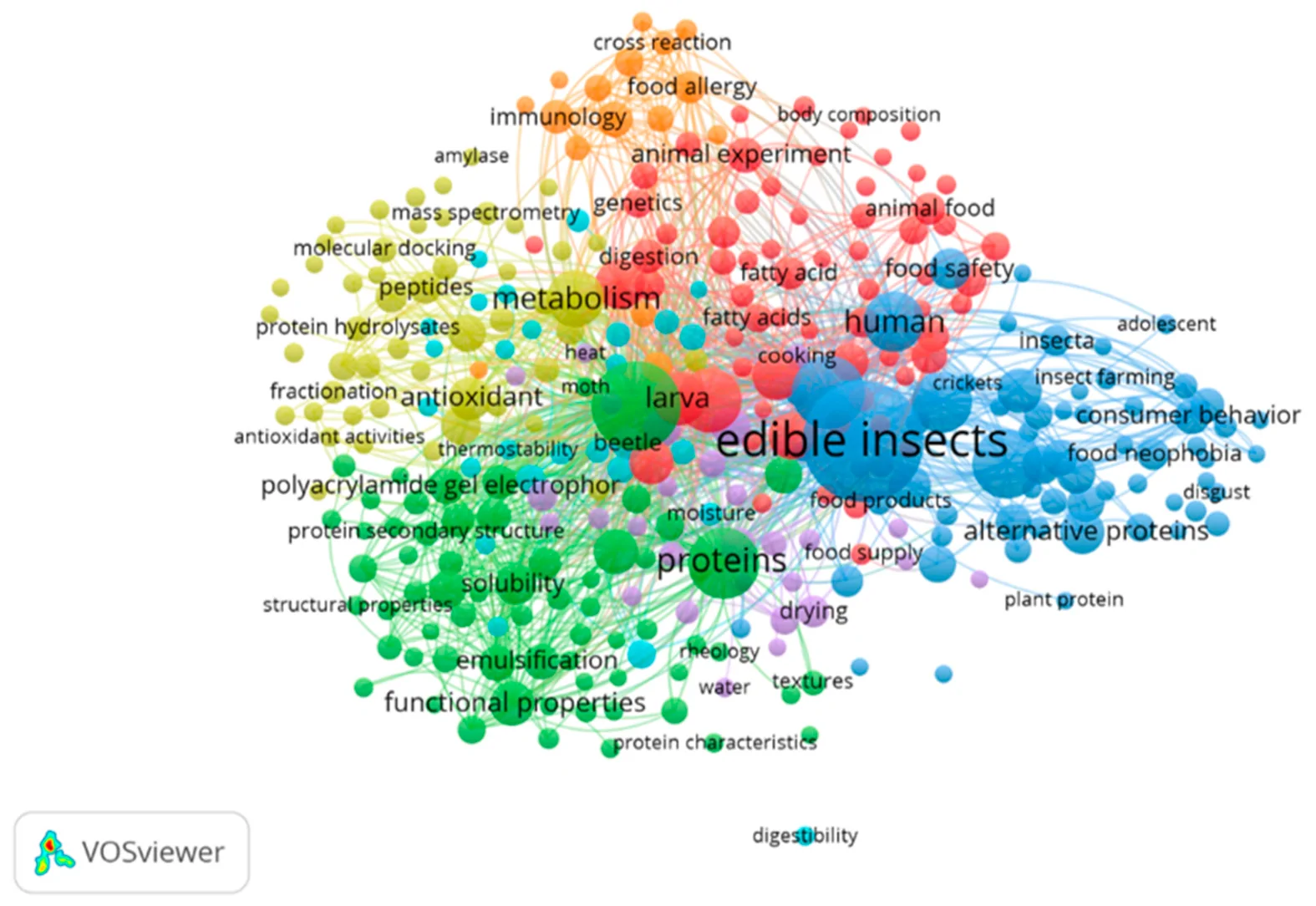
Keyword co-occurrence network showing the conceptual structure and major thematic clusters in edible insect-based product development research, 2016–2025 (317 keywords meeting the minimum occurrence threshold of 5; 14,221 co-occurrence links; VOSviewer clustering, resolution = 1.15). Node size represents keyword occurrence, edge thickness represents co-occurrence strength, and node colors denote cluster membership.

Figure 7 shows the keyword co-occurrence network and its seven thematic clusters, while Table 2 provides the detailed keyword composition and network metrics for each cluster. The corrected keyword co-occurrence analysis identified seven thematic clusters. The clusters were generated empirically from co-occurrence relationships, some keywords and concepts overlap across clusters. The labels are therefore interpretive summaries of dominant terms rather than predefined or mutually exclusive scientific categories.

The keyword co-occurrence network is anchored by highly connected terms such as “edible insects” (occurrence = 284), “insect protein” (129), and “proteins” (116). Their prominence indicates that nutritional and protein-related characterization remains structurally central to the indexed literature, while the seven-cluster structure demonstrates that this core is connected to consumer, processing, bioactive, allergenicity, and taxonomic dimensions [6,13].

The network also indicates different levels of conceptual integration. Broad terms such as “edible insects” and “insect protein” function as hubs linking multiple thematic areas, whereas narrower terms in the taxonomic/chemical and allergenicity clusters occupy more specialized positions. Such specialized positions indicate lower connectivity within the keyword network, not lower scientific importance; interpretation is therefore based on co-occurrence structure rather than visual prominence alone.

Table 2 presents the complete cluster composition and network metrics (TLS and occurrence) underlying the seven-cluster structure introduced above; detailed interpretation of each cluster is provided in Section 4.3. Table 3 complements this keyword-level view by quantifying how these same seven clusters are distributed across the 542-record corpus.

Beyond thematic coverage, citation-based visibility offers a complementary indicator of scholarly attention within the corpus, summarized in Table 4.

The ten most-cited records (Table 4) concentrate on recurring research problems linking fundamental characterization with practical food-system questions. Highly cited studies address nitrogen-to-protein conversion and nutrient composition, insect-enriched food formulation, insect-protein processing and functionality, consumer acceptance, allergenicity, and sustainability or regulatory aspects. This pattern suggests that studies connecting basic evidence to food quality, safety, processing, or adoption have attracted comparatively high scholarly visibility. Because citation counts are accumulated at the extraction date, they should be interpreted as indicators of historical scholarly attention rather than direct measures of current research momentum.

### 3.4. International Collaboration and Network Integration [53,54]

**Figure 8:**
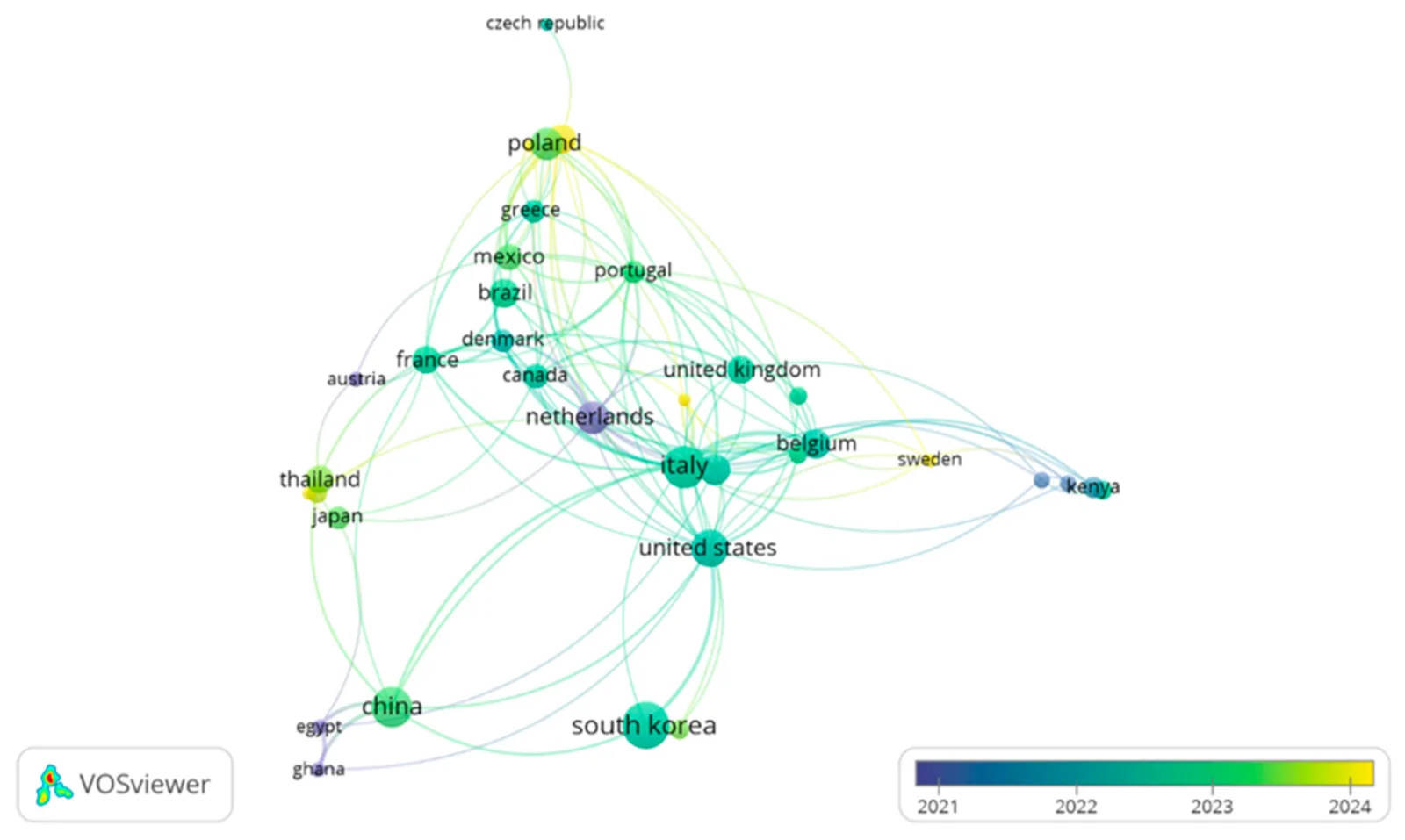
Country co-authorship overlay visualization for edible insect-based product development research (countries with ≥5 documents; edges with collaboration frequency ≥ 2 shown for legibility). Node size represents document output, edge thickness represents collaboration frequency, and node color represents the average publication year associated with each country, with later colors indicating more recent average publication years.

**Figure 9:**
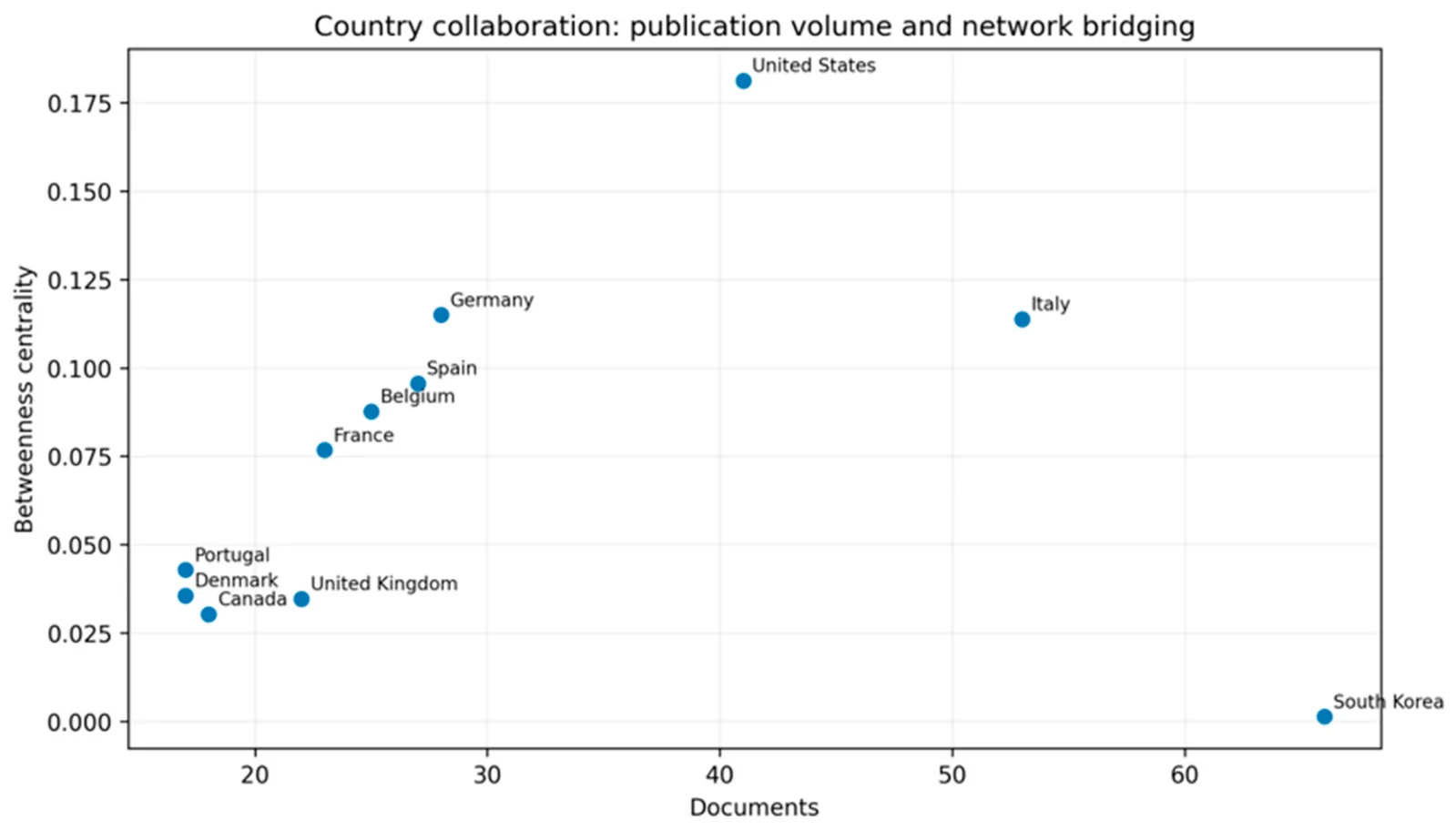
Quantitative country-collaboration metrics used to evaluate international network integration; structural bridging is assessed using betweenness centrality and Total Link Strength rather than visual inspection alone.

Figure 8 visualizes the country co-authorship network and its temporal overlay, whereas Figure 9 summarizes the quantitative relationship between publication volume and network bridging. The metric-based reconstruction shows that international collaboration is structured around a European core, including Italy, Belgium, Germany, the Netherlands, and Denmark. Table 5 provides quantitative support for the interpretation of structural bridging: the United States has the highest betweenness centrality (0.1813), followed by Germany (0.1150) and Italy (0.1138), whereas South Korea has very low betweenness (0.0013) despite the largest publication volume. Thus, terms such as “bridge” or “central” are used here only where supported by network measures rather than by visual inspection. The collaboration structure suggests that some countries connect otherwise separated research communities, while others generate substantial output with fewer international links.

South Korea—the single largest contributor by publication volume (66 documents)—has only 4 distinct international partner countries, a collaboration TLS of 9, and betweenness centrality of 0.0013. This indicates comparatively limited international-collaboration integration within the indexed network. Africa and Southeast Asia are also comparatively peripheral by document volume and collaboration-link count; most countries in these regions fell below the 5-document threshold for stable centrality estimation. This pattern should not be interpreted as evidence of limited local research activity or relevance. It may partly reflect database coverage, language and indexing structures, unequal access to internationally indexed publication platforms, differences in international co-authorship opportunities, and the fact that bibliometric databases do not capture all traditional or locally oriented research [40,55].

### 3.5. Evolution of Research Topics and Emerging Trends

**Figure 10:**
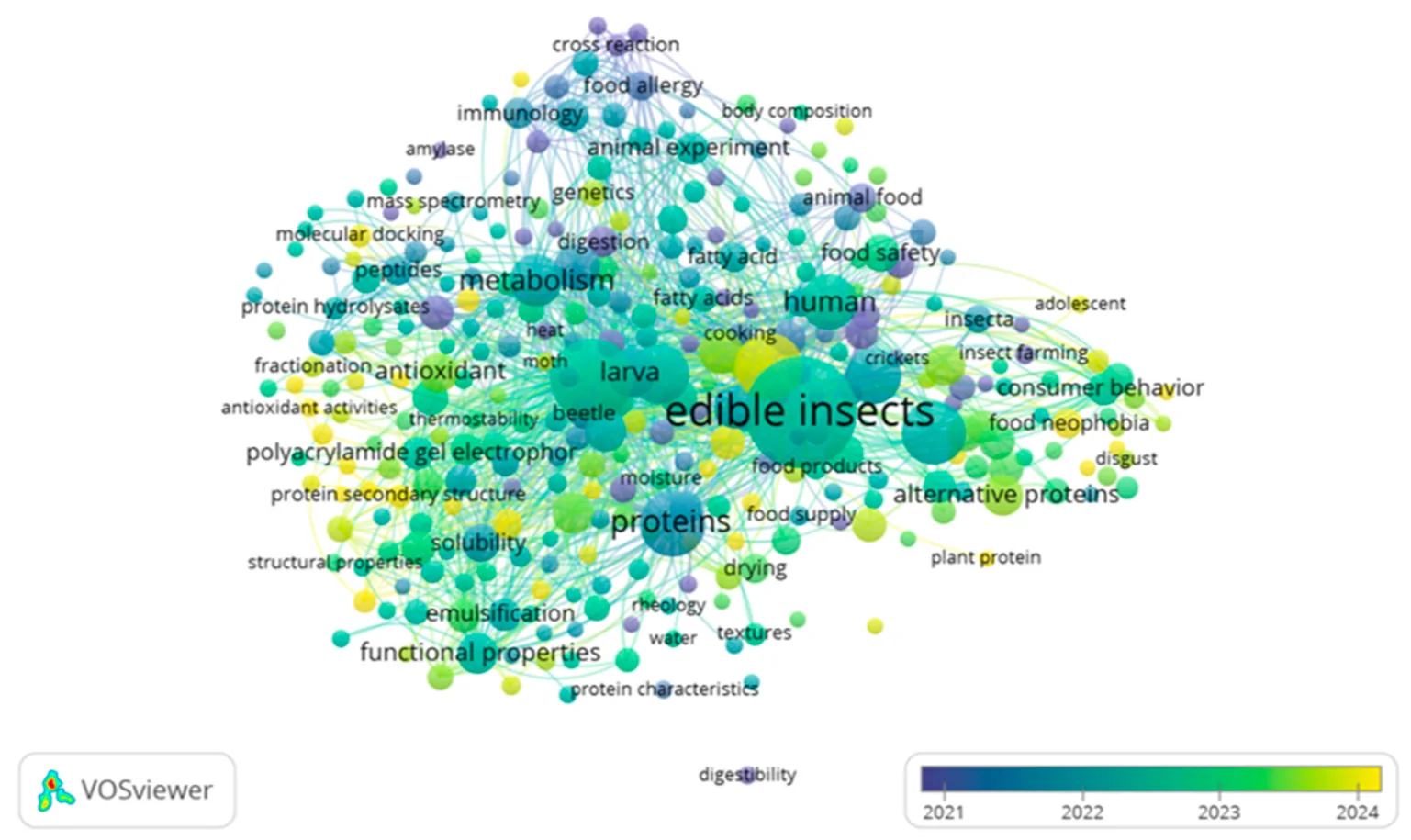
Keyword overlay visualization showing the average publication year associated with keywords in the edible insect-based product development corpus; later values indicate more recent average occurrence and should not automatically be interpreted as newly emerged research directions.

Figure 10 shows the keyword overlay visualization, which maps the average publication year associated with the keywords. Restricting to keywords with occurrence ≥ 10 (for statistical stability), the keywords with the most recent average occurrence year include “protein secondary structure” (avg. year 2024.4), “zeta potential” (2024.1), “food neophobia” (2024.0, *n* = 17 documents), “sustainable protein” (2023.9), “molecular docking” (2023.9), “insect-based food” (2023.8, *n* = 88), and “food security” (2023.7). The keywords with the earliest average occurrence year include “dietary protein” (2020.6), “allergenicity” (2020.7), “protein intake” (2020.8), “Diptera” (2020.9), and “hydrolysis” (2021.0). Notably, several very-high-occurrence, broadly used keywords—“edible insects” (avg. year 2022.6, *n* = 284), “insect protein” (2022.5, *n* = 129), “entomophagy” (2022.2, *n* = 75)—show only moderately recent average years despite substantial recent growth in absolute document count (Section 3.1), because their occurrences are distributed broadly across the full 2016–2025 window rather than concentrated in recent years. This distinction is important: it indicates that these terms are established, sustained themes of the field rather than newly emerging ones, whereas narrower-occurrence, more recent-average-year keywords such as “food neophobia” and “sustainable protein” more plausibly represent genuinely emerging or intensifying research emphases. We accordingly describe the field’s temporal evolution in terms of this quantitative recency measure rather than a purely visual “early vs. recent” reading of an overlay map.

## 4. Discussion

### 4.1. Positioning Edible Insects Within the Global Sustainable Protein Transition

The findings of this study substantiate the growing recognition of edible insects as a strategic component within the global transition toward sustainable protein systems. Rather than being positioned merely as an alternative food source, edible insects are increasingly conceptualized as integral elements of a diversified and resilient protein supply, consistent with global sustainability frameworks that advocate for dietary shifts toward environmentally efficient protein sources [2,4].

The observed disparities can plausibly arise from interacting structural factors rather than a single determinant. Research funding and analytical infrastructure can influence the ability to conduct advanced compositional, allergenicity, and protein-functionality studies; industrial investment can shape opportunities for product-development research; and international partnerships can expand access to specialized analytical facilities and publication networks. Regulatory certainty may also influence research and commercialization pathways in jurisdictions where edible insects are treated within established novel-food frameworks [15,29,56]. However, these mechanisms are contextual explanations supported by the literature rather than causal relationships demonstrated by this bibliometric analysis. Historical entomophagy and high insect biodiversity in Africa and Southeast Asia may sustain substantial local relevance even when publication visibility in Scopus is comparatively low [40,55]. Accordingly, publication productivity should be distinguished from local relevance, traditional knowledge, production capacity, and scientific influence.

### 4.2. Structural Determinants of Knowledge Production and Global Disparities

The country-level results (Section 3.2.1) and the network-metric results (Section 3.4, Table 5) together show a geographically and structurally uneven distribution of Scopus-indexed research activity that is more nuanced than publication counts alone reveal. We distinguish three related but distinct constructs: (i) publication volume (South Korea leads at 66 documents); (ii) international-collaboration integration, measured by network centrality (South Korea ranks among the lowest of major contributors, betweenness = 0.0013, versus the United States at 0.1813); and (iii) local research relevance, traditional ecological knowledge, and production capacity, which this bibliometric analysis cannot measure directly and which may be substantial in low-Scopus-output regions [57,58,59].

This decomposition reveals that “leading the field” can mean structurally different things: South Korea’s pattern is consistent with a well-resourced, government-supported, but comparatively domestically oriented national research program [38,60], whereas the United States, Germany, and Italy’s high betweenness centrality indicates their research communities function as connective infrastructure linking otherwise less-connected parts of the international network—plausibly reflecting, respectively, US cross-sector academia-industry-startup collaboration in alternative-protein innovation [48,61], and Germany and Italy’s position within coordinated European Union research-funding programs and the Novel Food regulatory pathway [15,62]. We present these as plausible, literature-consistent interpretations of a genuine structural pattern in the network data, rather than as causal claims directly tested by this bibliometric analysis. By contrast, research capacity in much of Africa and Southeast Asia is very likely constrained less by scientific interest—given extensive documented traditional entomophagy practice and insect biodiversity in these regions [40,55]—than by structural factors including comparatively limited competitive research-funding infrastructure, fewer opportunities for international co-authorship, and the English-language, Scopus-indexing biases discussed in Section 4.8.

### 4.3. Thematic Integration of Knowledge Clusters

The seven thematic clusters identified in Table 2 collectively describe the intellectual architecture of edible insect-based product research: (1) entomophagy, novel food, and consumer safety; (2) nutritional and compositional validation; (3) protein structural and functional properties; (4) taxon-specific protein sources; (5) bioactive compounds and enzymatic processing; (6) allergenicity and immunological safety; and (7) taxonomic and chemical characterization. These clusters were derived empirically from keyword co-occurrence rather than imposed as predefined conceptual categories. Accordingly, cluster labels should be interpreted as concise summaries of dominant co-occurring terms rather than mutually exclusive research categories.

Cluster 1 (edible insects, insect-based food, entomophagy, novel food, food safety, consumer attitude) is represented in 412 of 542 documents (76.0%) containing at least one of its top-10 keywords. This high coverage indicates that entomophagy, novel-food framing, and consumer/safety terminology are pervasive across the corpus, although the measure should be interpreted as keyword-based coverage rather than the proportion of publications devoted exclusively to this theme. A representative highly matched paper is an Italian consumer study on alternative protein sources (Meat Science, 2022, 40 citations).

Cluster 2 (*Tenebrio molitor*, larva, amino acids, nutritional value, protein intake, digestion) covers 166 documents (30.6%) and constitutes the foundational, compositional-validation layer of the field, reflecting species-specific nitrogen-to-protein conversion factors and digestibility studies that moved beyond crude protein content reporting [44,63,64]. The most keyword-representative paper in this cluster is a 2016 safety assessment of freeze-dried *Tenebrio molitor* larvae (Regulatory Toxicology and Pharmacology, 53 citations) [65,66,67,68].

Cluster 3 (protein content, polyacrylamide gel electrophoresis, solubility, functional properties, hydrophobicity) covers 112 documents (20.7%) and concerns the relationship between protein structural engineering and techno-functional performance; processing treatments such as defatting, thermal treatment, and alkaline extraction significantly alter protein conformation and thereby influence solubility, emulsification, and foaming capacity [38,69,70].

Cluster 4 (proteins, *Acheta domesticus*, Gryllidae, protein source, Caelifera, cricket) covers 191 documents (35.2%) and combines cricket/grasshopper taxa with generic protein source terminology, most plausibly because these orthopteran taxa are the most frequently studied insect protein sources after *Tenebrio molitor*, spanning processing and formulation studies including house cricket (*Acheta domesticus*) flour products [71] and grasshopper-based meat analogs [72].

Cluster 5 (antioxidant, hydrolysis, antioxidant activity, protein hydrolysate, peptides, pupa) covers 86 documents (15.9%) and reflects the bioactive-compound research frontier: enzymatic hydrolysis of insect proteins generates bioactive peptides with antioxidant and angiotensin-converting enzyme (ACE) inhibitory activity [13,17,73,74].

Cluster 6 (insect protein, allergen, immunology, food allergy, immunoglobulin E, allergenicity) covers 150 documents (27.7%) and addresses allergenicity and immunological safety; a representative paper is a 2018 review of insect food allergy and allergens (Molecular Immunology, 224 citations). Notably, this cluster is anchored by the generic “insect protein” keyword (the single highest-TLS keyword in the entire network, TLS = 1824) rather than by allergenicity-specific terms alone, suggesting that allergenicity research is discussed largely in relation to, rather than independently of, general protein source framing—itself a signal that allergenicity has not yet developed as fully independent a sub-literature as the compositional clusters (2–4).

Cluster 7 (beetle, Diptera, Coleoptera, chemical composition, *Hermetia illucens*, tryptophan) covers 76 documents (14.0%)—the smallest of the seven clusters—and centers on taxonomic classification and basic chemical characterization across a broader range of insect taxa beyond the dominant *Tenebrio molitor* and Orthoptera groups emphasized in Clusters 2 and 4. This cluster combines order- and species-level taxonomic terms (Diptera, Coleoptera, *Hermetia illucens*, *Alphitobius diaperinus*, *Gryllus bimaculatus*) with compositional descriptors such as moisture, chitin, and tryptophan content, reflecting foundational proximate-composition and taxonomic-identification studies that establish species-specific chemical profiles prior to further functional or safety evaluation. The comparatively low keyword-based coverage of this cluster suggests that taxonomic and chemical characterization research remains less consolidated than the compositional-validation literature centered on *Tenebrio molitor* (Cluster 2), indicating an opportunity for more systematic chemical profiling across the wider range of edible insect taxa represented in the corpus.

### 4.4. Consumer Acceptance and Product Format

Consumer acceptance is closely linked to product format, sensory experience, and the extent to which insect-derived ingredients are integrated into familiar foods. The high keyword-based coverage of Cluster 1 (76.0%; Table 3) indicates that entomophagy, consumer attitudes, novel food, and safety are pervasive across the corpus. However, co-occurrence prominence should not be interpreted as evidence of uniform consumer acceptance. Studies represented in this literature commonly distinguish between visibly whole insects and less visually salient ingredients such as powders, protein concentrates, or isolates incorporated into familiar matrices. Cricket-powder-enriched bread, for example, illustrates the use of a familiar bakery matrix in which insect flour is incorporated as an ingredient [71], while insect–plant protein meat analogs demonstrate another product-development route [72]. These examples suggest that product familiarity can interact with the visibility and sensory salience of insect ingredients [75,76,77].

Sensory attributes remain important determinants of acceptance. Texture, flavor, aroma, and color can influence both willingness to try and subsequent product evaluation, while perceived health and environmental benefits may provide additional motivation for some consumers [16,31,32,52,78]. Price and availability also affect the transition from stated interest to actual purchasing behavior, and cultural context and previous entomophagy experience can modify responses. Food neophobia and disgust may be particularly relevant among consumers without prior exposure to entomophagy. These factors indicate that product reformulation alone cannot be assumed to overcome sociocultural barriers; consumer segmentation and context-specific sensory research remain necessary.

The distinction between hypothetical willingness-to-try and repeat purchase is particularly important. Stated intentions obtained from surveys can identify attitudes and potential market segments but cannot establish sustained purchasing behavior under real market conditions. Accordingly, product development should combine sensory optimization with transparent communication, clear ingredient and allergen labeling, traceability, and market-based evaluation. Reducing the visual or sensory salience of insect ingredients may facilitate initial trial, but it should not be treated as a sufficient long-term strategy because informed choice and consumer trust remain essential. The bibliometric prominence of consumer-related terms in Cluster 1 therefore indicates a continuing research interface between food technology, sensory science, consumer behavior, and regulatory communication rather than a simple technological solution to acceptance.

### 4.5. Technological Maturation and Functional Food Development

The progression of themes from compositional characterization toward protein functionality, processing, and bioactive applications is consistent with the seven-cluster structure identified in Table 2 and the temporal patterns in Figure 10. Processing technologies are important because they determine not only ingredient functionality but also safety, digestibility, sensory quality, and the feasibility of incorporating insect-derived materials into specific food matrices. Drying and thermal processing can improve microbial stability and storage characteristics, but treatment intensity may alter nutrient retention, protein conformation, solubility, and other functional properties [79]. Defatting, particularly of lipid-rich insects such as *Hermetia illucens*, can modify the physicochemical characteristics of the resulting protein fraction and thereby affect emulsification, foaming, and solubility [80,81,82,83,84,85].

Protein extraction and fractionation provide routes for concentrating or modifying soluble proteins. Wet or dry fractionation and alkaline extraction can increase the availability of protein-rich fractions, although pH, temperature, solvent conditions, and subsequent drying influence protein structure and techno-functional performance [69,86]. Enzymatic hydrolysis can produce lower-molecular-weight peptides with reported antioxidant or angiotensin-converting enzyme (ACE)-inhibitory activities [17,73], but enzyme cost, reaction control, and downstream separation remain relevant to scale-up. Fermentation may modify flavor, digestibility, and functional characteristics through biochemical transformation of insect substrates, while incorporation into bread and meat analogs demonstrates how the final food matrix influences the practical value of the processed ingredient [71,72,87].

Additional technologies such as extrusion and texturization are relevant where the objective is to transform insect proteins into structured products with meat-like or snack-like textures. Ultrasonication and high-pressure processing are promising approaches for modifying protein structure and potentially improving functional properties, but their insect-specific effects, energy requirements, cost, and scalability require systematic comparison. Across these approaches, processing conditions should be evaluated together with species, rearing substrate, pretreatment, and food matrix because these factors can alter nutritional, sensory, and functional outcomes [88].

Technological maturation therefore involves trade-offs rather than a simple progression toward more intensive processing. Excessive heating can promote protein denaturation and reduce solubility; lipid-rich materials may be susceptible to oxidation and associated off-flavors or darkening; and intensive drying or fractionation can increase energy demand and processing costs. Freeze-drying may preserve high-quality dried material but can be more energy-intensive than conventional convective drying. Future studies should compare processing routes using standardized quality, safety, sensory, digestibility, and techno-economic endpoints so that technological feasibility can be distinguished from industrial readiness [89].

### 4.6. Food Safety, Allergenicity, and Regulatory Considerations

Safety considerations remain multidimensional and should not be generalized across all edible insect species, substrates, processes, and product forms. Cluster 6 (27.7% keyword-based coverage; Table 3) captures allergenicity and immunological safety, including concerns about structural similarities between insect allergenic proteins and known crustacean and dust-mite allergens and the resulting possibility of cross-reactivity in sensitized individuals [14]. The prominence of the generic “insect protein” keyword within this cluster also indicates that allergenicity research remains closely embedded in the broader protein source literature rather than forming a completely independent research stream. Microbial risks depend on rearing substrate, storage conditions, and processing method [79], while chemical and environmental contaminants, including potential heavy metals, pesticides, and veterinary residues, may also be substrate-dependent [90,91,92].

Processing can affect safety as well as functionality. Thermal treatment may reduce microbial hazards, but the effects of heat, hydrolysis, fermentation, and other treatments on allergenicity are not fully characterized across species and product forms. Regulatory assessments therefore need to consider the specific insect species, substrate, processing conditions, and intended food use. Within the European Union, assessments of specific edible insect species and products illustrate this product-specific approach [29,93]. Differences among jurisdictions and the continuing need for allergen disclosure, traceability, and consistent safety evaluation remain relevant to international commercialization [94].

The bibliometric prominence of safety-related terms, together with the comparatively less independent allergenicity vocabulary within the network, indicates a need for more harmonized approaches to allergen characterization and risk assessment. Future work should connect compositional and protein-characterization methods with validated allergenicity, microbial, chemical-contaminant, and toxicological endpoints, while also considering how processing and food-matrix effects alter exposure. These priorities should be treated as research needs indicated by the literature rather than as causal conclusions established by the bibliometric network.

### 4.7. Research Priorities and Future Directions

The bibliometric findings support several research priorities directly. First, allergenicity and immunological-safety research warrants greater methodological harmonization because the allergenicity-related cluster is smaller and less independently defined than the major compositional and protein-functionality clusters. Second, Africa and Southeast Asia remain less represented in the Scopus-indexed publication and collaboration network; future work should therefore strengthen research capacity, regional visibility, and equitable international collaboration without equating lower indexed output with lower local research relevance. Third, South Korea combines the highest publication volume with comparatively limited international-network integration, indicating an opportunity to broaden cross-national collaboration around its substantial research output. Fourth, the limited representation of scale-up and techno-economic terminology in the network points to a need for more systematic evaluation of industrial feasibility [95].

Broader priorities identified through the literature include standardized species and processing terminology; harmonized methods for protein quality, digestibility, and bioavailability; consumer segmentation and real-market purchasing studies that move beyond hypothetical willingness-to-try; comparative life cycle assessment; evaluation of processing side-streams; animal-welfare and ethical considerations in mass rearing; traceability and authenticity systems; regulatory convergence across jurisdictions; and equitable inclusion of traditional knowledge-holders and researchers from insect-consuming regions in international collaborations. Distinguishing these literature-informed priorities from those directly visible in the bibliometric structure helps avoid treating the network itself as causal evidence.

### 4.8. Limitations

This study has several limitations. First, it used Scopus as a single database and English-language records, so regional-language and non-Scopus-indexed research may be underrepresented, particularly in Africa, Southeast Asia, and other insect-consuming regions. Second, the original Boolean query contains mixed OR and AND operators, creating potential false-positive and false-negative retrieval. The post hoc record-level audit applied a conservative rule requiring at least one insect-food term and one food/product term; 518 of 542 records (95.6%) met this rule and 24 (4.4%) did not. The audit was a retrieval-risk check rather than an eligibility filter, and the original 542-record corpus was retained unchanged. The original Scopus query was not rerun during this revision, so a new-query overlap analysis was not performed. Third, no independent dual-author title/abstract screening was conducted, and the corpus includes 536 records classified by Scopus as Articles and 6 Conference Papers; Scopus document type does not by itself establish that every Article is primary research. Fourth, citation comparisons across publication years are affected by unequal citation windows, particularly for the 2025 cohort. Fifth, bibliometric results represent the published Scopus-indexed literature and cannot directly capture unpublished industrial research and development, traditional practice, local research relevance, or actual commercial-scale progress. Sixth, keyword indexing and normalization affect co-occurrence structures; although an explicit thesaurus and stop-term procedure were applied, the resulting clusters remain dependent on the terminology present in the source records. Seventh, network visualization and clustering depend on software settings and layout parameters, and the historical VOSviewer version used for the original analysis was not retained. Finally, keyword co-occurrence and centrality identify associations and network structure but cannot by themselves establish scientific consensus, causal relationships, technological maturity, commercial feasibility, or consumer acceptance. Such conclusions require direct evidence from representative primary studies, systematic reviews, sensory or human studies, techno-economic assessments, and market research.

## 5. Conclusions

Network-metric analysis of country-level collaboration provides a more structurally nuanced picture than publication counts alone. The United States, Germany, and Italy occupy the strongest bridging positions in the international collaboration network, whereas South Korea—despite having the highest publication volume—shows comparatively limited international-collaboration integration. Africa and Southeast Asia remain comparatively peripheral in the Scopus-indexed network; this pattern may partly reflect database coverage, English-language and indexing structures, and unequal international collaboration opportunities rather than an absence of local research activity or relevance.

Based on the bibliometric evidence, the main research gaps concern methodologically harmonized allergenicity assessment, broader international collaboration involving underrepresented regions and under-networked high-output countries, and more systematic evaluation of processing scale-up and techno-economic feasibility. These priorities represent directions indicated by the indexed literature and should be evaluated through direct empirical, technological, safety, and market evidence.

## Figures and Tables

**Table 1:** Ten most productive authors in edible insect-based product development research (2016–2025).

Author	Documents	Total Citations	Primary Affiliation (Scopus Author ID-Matched)
Choi, Yun-sang	25	738	Korea Food Research Institute
Kim, Tae-kyung	21	670	Korea Food Research Institute
Yong, Hae In	14	571	Korea Food Research Institute
Fogliano, Vincenzo	12	1033	Wageningen University & Research
Lakemond, Catriona M.M.	10	1007	Wageningen University & Research
Lee, Jaehoon	10	211	Korea Food Research Institute
Jang, Haewon	10	217	Sungshin Women’s University
Jung, Samooel	9	396	Chungnam National University
Kang, Mincheol	9	236	Korea Food Research Institute
Mishyna, Maryia Yu	8	253	Wageningen University & Research

**Table 2:** Main keyword clusters identified in edible insect-based product development research (2016–2025), based on VOSviewer clustering (resolution = 1.15) of a keyword co-occurrence network built directly from the Scopus export with an explicit merge thesaurus applied (see Section Bibliometric Network Analysis Parameters). TLS = Total Link Strength, the sum of co-occurrence link weights of a given keyword with all other keywords in the network. Occ. = Occurrence, the number of documents (out of 542) in which the keyword appears. Network totals: 317 keywords and 14,221 links met the minimum-occurrence threshold of 5 across the full network. Bold text denotes thematic cluster labels.

Cluster 1—Entomophagy, Novel Food & Consumer Safety		
Keyword	TLS	Occ.
edible insects	2114	284
insect-based food	1179	88
insects	711	71
entomophagy	428	75
alternative protein	341	60
nutrition	304	29
food safety	278	27
novel food	254	39
consumer attitude	234	20
meat	229	17
**Cluster 2—Nutritional & Compositional Validation**		
**Keyword**	**TLS**	**Occ.**
*Tenebrio molitor*	837	66
Tenebrio	678	41
larva	672	47
amino acids	632	42
nutritional value	520	36
protein intake	377	20
digestion	373	18
animal experiment	353	21
dietary protein	325	18
animal tissue	268	12
**Cluster 3—Protein Structural & Functional Properties**		
**Keyword**	**TLS**	**Occ.**
protein content	697	40
polyacrylamide gel electrophoresis	506	20
solubility	491	27
functional properties	475	34
hydrophobicity	465	22
particle size	399	20
isolation and purification	396	18
physical chemistry	383	17
emulsification	365	21
extraction	331	19
**Cluster 4—Taxon-Specific Protein Sources**		
**Keyword**	**TLS**	**Occ.**
proteins	1395	116
*Acheta domesticus*	520	42
Gryllidae	422	25
protein source	287	25
Caelifera	219	12
flour	202	14
cricket	201	21
iron	178	11
food ingredient	172	9
food products	169	13
**Cluster 5—Bioactive Compounds & Enzymatic Processing**		
**Keyword**	**TLS**	**Occ.**
antioxidant	431	32
hydrolysis	341	23
antioxidant activity	329	25
protein hydrolysate	258	15
peptides	252	17
pupa	237	14
molecular weight	233	11
mice	218	10
mouse	218	10
enzymatic hydrolysis	203	17
**Cluster 6—Allergenicity & Immunological Safety**		
**Keyword**	**TLS**	**Occ.**
insect protein	1824	129
allergen	319	22
immunology	311	18
food allergy	278	17
food hypersensitivity	226	14
immunoglobulin E	208	12
*Locusta migratoria*	201	15
*Bombyx mori*	192	11
allergenicity	168	10
heat treatment	159	12
**Cluster 7—Taxonomic & Chemical Characterization**		
**Keyword**	**TLS**	**Occ.**
beetle	311	17
Diptera	243	15
Coleoptera	221	12
chemical composition	202	14
*Hermetia illucens*	190	18
tryptophan	171	7
*Alphitobius diaperinus*	165	10
moisture	164	9
*Gryllus bimaculatus*	158	10
chitin	151	10

**Table 3:** Publication coverage of the seven thematic clusters. * Coverage denotes the number and percentage of the 542 records containing at least one of the top-10 keywords assigned to the corresponding cluster; categories are non-mutually exclusive, so percentages may sum to more than 100%.

Cluster	Interpretive Theme	Publication Coverage *	Share *
1	Entomophagy, novel food & consumer safety	412	76.0%
2	Nutritional & compositional validation	166	30.6%
3	Protein structural & functional properties	112	20.7%
4	Taxon-specific protein sources	191	35.2%
5	Bioactive compounds & enzymatic processing	86	15.9%
6	Allergenicity & immunological safety	150	27.7%
7	Taxonomic & chemical characterization	76	14.0%

**Table 4:** Ten most-cited records in the Scopus export. * Citation counts are total Scopus “Cited by” counts at the 8 February 2026 extraction date and therefore reflect unequal citation windows across publication years.

Rank	First Author/Year	Article	Journal	Cited by *
1	Janssen, Renske H. (57196713726) (2017) [44]	Nitrogen-to-Protein Conversion Factors for Three Edible Insects: *Tenebrio molitor*, *Alphitobius diaperinus*, and *Hermetia illucens*	Journal of Agricultural and Food Chemistry	719
2	Liu, Xiu (57195334380) (2017) [45]	Dynamic changes of nutrient composition throughout the entire life cycle of black soldier fly	PLoS ONE	349
3	van Huis, Arnold (7003540640) (2016) [10]	Edible insects are the future?	Proceedings of the Nutrition Society	290
4	Severini, Carla (7005669524) (2018) [46]	On printability, quality and nutritional properties of 3D printed cereal based snacks enriched with edible insects	Food Research International	270
5	Hadi, Joshua Lemuel (56383017400) (2021) [47]	Safety of alternative proteins: Technological, environmental and regulatory aspects of cultured meat, plant-based meat, insect protein and single-cell protein	Foods	265
6	Sexton, Alexandra E. (57193759277) (2019) [48]	Framing the future of food: The contested promises of alternative proteins	Environment and Planning E: Nature and Space	242
7	Kim, Hyun-wook (57843852100) (2016) [49]	Pre-treated mealworm larvae and silkworm pupae as a novel protein ingredient in emulsion sausages	Innovative Food Science and Emerging Technologies	237
8	Halloran, Afton (55891406800) (2016) [50]	Life cycle assessment of edible insects for food protein: A review	Agronomy for Sustainable Development	228
9	de Gier, Steffie (57191418122) (2018) [51]	Insect (food) allergy and allergens	Molecular Immunology	224
10	Orsi, Luigi (55499248300) (2019) [52]	Eating edible insects as sustainable food? Exploring the determinants of consumer acceptance in Germany	Food Research International	224

**Table 5:** Country-level collaboration network metrics for edible insect-based product development research (countries with ≥5 documents). TLS = Total Link Strength, the sum of collaboration-link weights with other countries. Betweenness centrality quantifies each country’s structural bridging role between otherwise less-connected parts of the network.

Country	Documents	Partner Countries	TLS (Collab. Links)	Betweenness Centrality
United States	41	20	33	0.1813
Germany	28	13	28	0.1150
Italy	53	19	56	0.1138
Spain	27	16	26	0.0956
Belgium	25	14	30	0.0877
France	23	12	20	0.0767
Portugal	17	14	26	0.0428
United Kingdom	22	11	15	0.0347
Denmark	17	11	30	0.0356
Canada	18	11	14	0.0303
South Korea	66	4	9	0.0013

## Data Availability

The bibliographic records analyzed in this study were retrieved from Scopus and are subject to Scopus licensing and redistribution restrictions. The record-level export therefore cannot be publicly redistributed by the authors. The controlled topical-verification vocabulary and acceptance rule, terminology thesaurus and stop-term principles, analysis workflow and parameters, and derived summary results that can be shared without redistributing licensed records are provided in the Supplementary Reproducibility Information. Additional derived materials may be made available by the corresponding author upon reasonable request, subject to database licensing terms.

## Supplementary Materials

The following supporting information can be downloaded at: https://www.mdpi.com/article/10.3390/insects17101005/s1, Supplementary File S1: Supplementary Reproducibility Information, including the controlled topical-verification vocabulary and acceptance rule, terminology-normalization thesaurus and stop-term list, analysis workflow and parameters, record-level audit, and derived summary results.
